# Suppression of store-operated calcium entry causes dilated cardiomyopathy of the *Drosophila* heart

**DOI:** 10.1242/bio.049999

**Published:** 2020-03-11

**Authors:** Courtney E. Petersen, Matthew J. Wolf, Jeremy T. Smyth

**Affiliations:** 1 Graduate Program in Molecular and Cellular Biology, Uniformed Services University of the Health Sciences, F. Edward Hébert School of Medicine, Bethesda, MD 20814, USA; 2 Division of Cardiovascular Medicine, Department of Medicine, The University of Virginia School of Medicine, Charlottesville, VA 22908, USA; 3 Department of Anatomy, Physiology, and Genetics, Uniformed Services University of the Health Sciences, F. Edward Hébert School of Medicine, Bethesda, MD 20814, USA

**Keywords:** Cardiomyopathy, Cardiac, Store-operated calcium entry, STIM, Orai, *Drosophila*

## Abstract

Store-operated Ca^2+^ entry (SOCE) is an essential Ca^2+^ signaling mechanism present in most animal cells. SOCE refers to Ca^2+^ influx that is activated by depletion of sarco/endoplasmic reticulum (S/ER) Ca^2+^ stores. The main components of SOCE are STIM and Orai. STIM proteins function as S/ER Ca^2+^ sensors, and upon S/ER Ca^2+^ depletion STIM rearranges to S/ER-plasma membrane junctions and activates Orai Ca^2+^ influx channels. Studies have implicated SOCE in cardiac hypertrophy pathogenesis, but SOCE's role in normal heart physiology remains poorly understood. We therefore analyzed heart-specific SOCE function in *Drosophila*, a powerful animal model of cardiac physiology. We show that heart-specific suppression of *Stim* and *Orai* in larvae and adults resulted in reduced contractility consistent with dilated cardiomyopathy. Myofibers were also highly disorganized in *Stim* and *Orai* RNAi hearts, reflecting possible decompensation or upregulated stress signaling. Furthermore, we show that reduced heart function due to SOCE suppression adversely affected animal viability, as heart specific *Stim* and *Orai* RNAi animals exhibited significant delays in post-embryonic development and adults died earlier than controls. Collectively, our results demonstrate that SOCE is essential for physiological heart function, and establish *Drosophila* as an important model for understanding the role of SOCE in cardiac pathophysiology.

## Abstract (precis)

**Summary:** Ca^2+^ dysregulation in cardiomyocytes is a common feature of cardiomyopathy and heart failure pathogenesis. Here we demonstrate in *Drosophila melanogaster* that store-operated Ca^2+^ entry is essential for physiological heart function.

## INTRODUCTION

Cardiomyopathies are a major cause of morbidity and mortality throughout the western world, with current treatment options limited to palliative pharmacological or invasive therapy (McKenna et al., 2017). The discovery of curative treatments depends on a thorough understanding of the molecular mechanisms that govern the onset and progression of cardiac pathophysiology. Significantly, irregularities in cardiomyocyte calcium (Ca^2+^) homeostasis are a major contributing factor to cardiomyopathy and heart failure pathogenesis, and targeting Ca^2+^ signaling mechanisms may therefore be an important approach to novel therapeutic development (Abraham and Wolf, 2013; Kranias and Bers, 2007; Limas et al., 1987; MacLeod, 2016 preprint; Tham et al., 2015).

The role of Ca^2+^ in the process of excitation-contraction (E-C) coupling, which drives cardiomyocyte contractility, is well established. In E-C coupling, membrane depolarization opens L-type voltage gated Ca^2+^ channels, generating localized Ca^2+^ elevations that activate ryanodine receptors (RyRs) in the sarcoplasmic reticulum (SR). Release of SR Ca^2+^ via RyRs results in a large cytoplasmic Ca^2+^ pulse that drives acto-myosin contractility (Bers, 2002). In addition to E-C coupling, Ca^2+^ is also an important regulator of cardiomyocyte signaling pathways, such as those that control differentiation, cell growth and pathological remodeling (Vega et al., 2003). Maintenance of Ca^2+^ homeostasis, including SR Ca^2+^ stores, is therefore essential to multiple aspects of cardiomyocyte physiology. Store-operated Ca^2+^ entry (SOCE) is a process that plays a major role in maintaining cellular Ca^2+^ homeostasis, as it couples the influx of extracellular Ca^2+^ to the depletion of sarco/endoplasmic (S/ER) Ca^2+^ stores. Ca^2+^ that enters the cell via SOCE can be pumped back into the S/ER to replenish depleted stores and restore S/ER Ca^2+^ homeostasis. Importantly, despite the prominent role for SR Ca^2+^ stores in cardiomyocyte physiology, the functions of SOCE in cardiomyocytes and overall cardiac physiology are poorly understood.

The main components of the SOCE pathway are STIM (stromal interacting molecule) and Orai. STIM is a single-pass transmembrane protein that serves as an S/ER Ca^2+^ sensor via its N-terminal EF-hand domain, and Orai is a SOCE pore forming channel subunit in the plasma membrane (Putney, 2011, 2018). In response to S/ER Ca^2+^ store depletion, STIM undergoes a large conformational change that results in oligomerization and exposure of a cytoplasmic Orai activating domain. Oligomerized STIM then translocates to S/ER-plasma membrane junctions where it interacts with and activates Orai to induce Ca^2+^ influx (Putney, 2018; Smyth et al., 2010). In mammals, there are two STIM isoforms, STIM1 and STIM2, and three Orai isoforms (Orai1–3), with STIM1 and Orai1 exhibiting the widest functional distribution across mammalian cell and tissue types.

Numerous studies strongly suggest that SOCE contributes to the pathogenesis of pathological cardiac hypertrophy, whereby heart muscle mass increases in response to stressors such as hypertension or valve dysfunctions. For example, induction of cardiac hypertrophy by pressure overload results in upregulation of STIM1 and Orai1 expression in mouse cardiac tissue, and cardiomyocyte-specific suppression of STIM1 and Orai1 attenuates the hypertrophic response (Benard et al., 2016; Hulot et al., 2011; Luo et al., 2012; Parks et al., 2016). Similarly, pharmacological induction of cardiac hypertrophy by phenylephrine and endothelin-1 is attenuated by suppression of STIM1 or Orai1 in rodent cardiomyocytes (Hulot et al., 2011; Luo et al., 2012; Voelkers et al., 2010). Enhanced SOCE in response to pathological stimuli likely activates the calcineurin-nuclear factor of activated T-cells (NFAT) signaling axis, which is essential for reactivation of developmental gene expression and promotion of cardiomyocyte growth (Hulot et al., 2011; Molkentin et al., 1998; Schulz and Yutzey, 2004; Wilkins et al., 2004).

In light of this strong evidence that enhanced SOCE can drive pathological responses in cardiomyocytes, an important question remains: what is the role of SOCE in healthy cardiomyocytes and physiological heart function? To this end, two independent studies have shown that cardiomyocyte restricted STIM1 deletion in mice results in marked left ventricular dilation and reduced ejection fraction in adult hearts (Collins et al., 2014; Parks et al., 2016). Decreased cardiac function was concomitant with indications of ER stress and changes in cardiomyocyte mitochondrial morphology (Collins et al., 2014), as well as altered contractile Ca^2+^ transients and myofibril organization (Parks et al., 2016). Additionally, Orai1 suppression in zebrafish embryos resulted in reduced fractional shortening and severe heart failure (Volkers et al., 2012). These results support the conclusion that SOCE is essential for normal cardiac physiology. Importantly though, the specific cellular processes that are regulated by SOCE in cardiomyocytes remain unknown. It is also unclear whether these results reflect full suppression of SOCE activity, because functional contributions by other STIM and Orai isoforms cannot be ruled out in these vertebrate models. The goal of our current study was to address these important questions by testing the role of SOCE in *Drosophila melanogaster* heart function, a valuable animal model in which powerful genetic tools can be integrated with *in vivo* analyses of cardiomyocyte physiology and overall heart function.

The *Drosophila* heart is a muscular tube of cardiomyocytes that runs along the dorsal midline of the animal. Its primary function is to pump hemolymph, a plasma-like fluid, throughout the body in an open circulatory system (Rotstein and Paululat, 2016). Importantly, the contractile physiology of the *Drosophila* heart, including cardiomyocyte Ca^2+^ transport mechanisms and sarcomere composition, is highly conserved with mammals (Lin et al., 2011; Ocorr et al., 2007), and the genetic and functional bases of many cardiomyopathies can be readily modeled and analyzed in flies (Piazza and Wessells, 2011). Simplified genetics is another important advantage of *Drosophila* over other animal models. In particular, *Drosophila* express single isoforms of *Stim* and *Orai*, thus precluding complications of functional overlap between multiple STIM and Orai isoforms encountered with vertebrate models. Taking advantage of the strengths of the *Drosophila* model, we demonstrate that animals with heart-specific suppression of the key SOCE pathway components, *Stim* and *Orai*, exhibit dilated cardiomyopathy characterized by enlarged end-diastolic and end-systolic dimensions and decreased fractional shortening. Myofibrils were highly disorganized and loosely spaced in *Stim* and *Orai* suppressed hearts, further consistent with disrupted contractile physiology. SOCE-suppressed animals also exhibited significantly delayed post-embryonic development and died earlier than controls, suggesting pathological impairment of cardiac function. Our results, as well as those from other animal models, demonstrate that SOCE has highly conserved, essential roles in supporting physiological heart function, and lay the groundwork for future studies using *Drosophila* to mechanistically define SOCE functions in the heart.

## RESULTS

### *Stim* and *Orai* suppression results in dilated cardiomyopathy

*Drosophila Stim* and *Orai* loss-of-function mutants fail to grow properly and die as second or third instar larvae (Pathak et al., 2017), limiting their use in analysis of heart function. We therefore used inducible RNAi to suppress *Stim* and *Orai* expression specifically in the heart and avoid the systemic effects of global SOCE suppression. Efficacy of the RNAi constructs was tested by expressing them ubiquitously in the whole animal and analyzing transcript levels and phenotypes. *Stim* and *Orai* RNAi driven by the ubiquitous *act-GAL4* driver suppressed *Stim* and *Orai* mRNA expression in whole first instar larvae by 72.67±3.18% and 80±2.65% (mean±s.e.m.), respectively, compared to non-targeting RNAi controls, demonstrating potent knockdown of the targeted transcripts (Fig. S1A). Ubiquitous expression of *Stim* and *Orai* RNAi also resulted in reduced growth and larval lethality (Fig. S1B–F) similar to loss-of-function mutants (Pathak et al., 2017), suggesting specific knockdown of the targeted gene products with few to no off-target effects. We also tested several other publicly available *Stim* and *Orai* RNAi *Drosophila* strains, but found that they were ineffective at suppressing *Stim* and *Orai* transcript levels, respectively (data not shown).

**Fig. 1.**
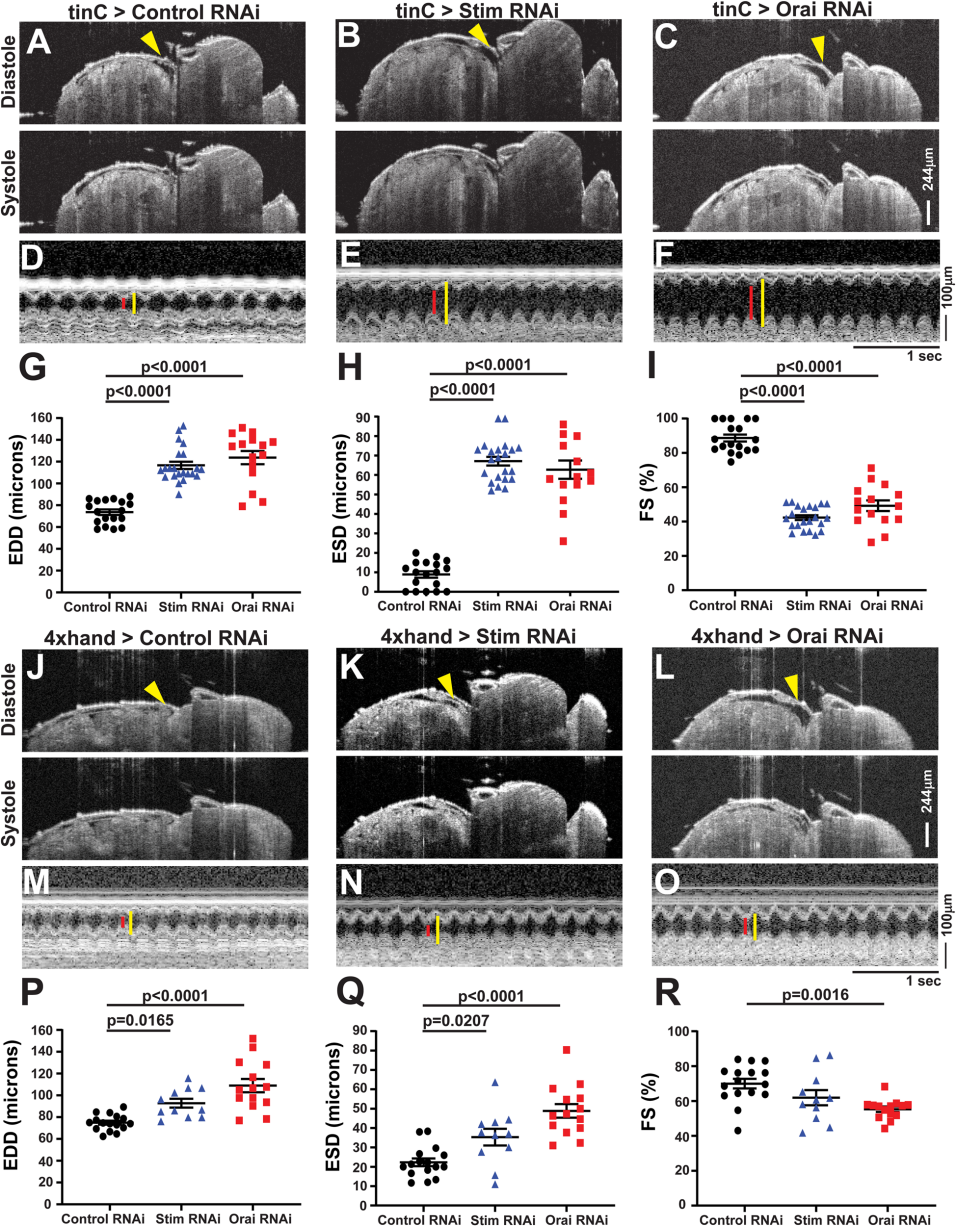
**Heart specific *Stim* and *Orai* suppression results in dilated cardiomyopathy in adults.** (A–C) Representative OCT longitudinal B-mode images of 5-day-old adult male flies with *tinC-GAL4* driven non-targeting control, *Stim* and *Orai* RNAi during diastole (upper panels) and systole (lower panels). Arrowheads indicate the heart in diastole images. (D–F) Representative M-mode recordings from *tinC-GAL4* driven non-targeting control, *Stim* and *Orai* RNAi with red lines depicting systole and yellow lines diastole. EDD (G), ESD (H), and FS (I) were calculated from M-mode recordings of 5-day-old males, and each symbol represents a measurement from a single animal. Bars indicate mean±s.e.m., and *P*-values were calculated from one-way ANOVA with Tukey's Multiple Comparison. (J–L) Representative OCT longitudinal B-mode images of 5-day-old adult male flies with *4xhand-GAL4* driven non-targeting control, *Stim* and *Orai* RNAi during diastole (upper panels) and systole (lower panels). Arrowheads indicate the heart in diastole images. (M–O) Representative M-mode recordings from *4xhand-GAL4* driven non-targeting control, *Stim* and *Orai* RNAi with red lines depicting systole and yellow lines diastole. EDD (P), ESD (Q), and FS (R) were calculated from M-mode recordings of 5-day-old adult males, and each symbol represents a measurement from a single animal. Bars indicate mean±s.e.m., and *P*-values were calculated from one-way ANOVA with Tukey's Multiple Comparisons Test.

We next evaluated heart contractility using optical coherence tomography (OCT) in fully intact, non-anesthetized animals with heart specific *Stim* and *Orai* suppression. Adult males with heart-specific *tinC-GAL4* driven *Stim* and *Orai* RNAi exhibited significantly increased end-diastolic dimensions (EDD) and a more pronounced increase in end-systolic dimensions (ESD) as compared to non-targeting RNAi controls (Fig. 1A–H). Based on these measures, the calculated fractional shortening (FS), a direct measure of the contractile strength of the heart, was 88.73±2.04% in non-targeting control hearts but was significantly reduced to 42.28±1.4% and 49.21±3.11% in *Stim* and *Orai* RNAi hearts, respectively (Fig. 1I; mean±s.e.m., *P*<0.001). Importantly, enlarged ESD and EDD and reduced FS in *Stim* and *Orai* RNAi animals are consistent with dilated cardiomyopathy. Similar results were also observed using a second heart specific driver, *4xhand-GAL4* (Fig. 1J–R). Animals with *4xhand-GAL4* driven *Stim* and *Orai* RNAi again exhibited significantly larger EDD and ESD compared to non-targeting RNAi controls, and this resulted in significantly reduced FS in *Orai* RNAi animals. The decrease in FS was not significantly different in animals with *4xhand-GAL4* driven *Stim* RNAi, likely due to less efficient knockdown of *Stim* compared to *Orai* expression (see Fig. S1A). Neither *tinC-GAL4* nor *4xhand-GAL4* driven *Stim* and *Orai* RNAi significantly altered heart rates or caused notable arrhythmias compared to controls (Fig. S2A,B). Overall, heart-specific suppression of *Stim* and *Orai* driven by two independent heart-specific drivers resulted in dilated cardiomyopathy, suggesting an essential function for SOCE in adult *Drosophila* cardiac function.

**Fig. 2.**
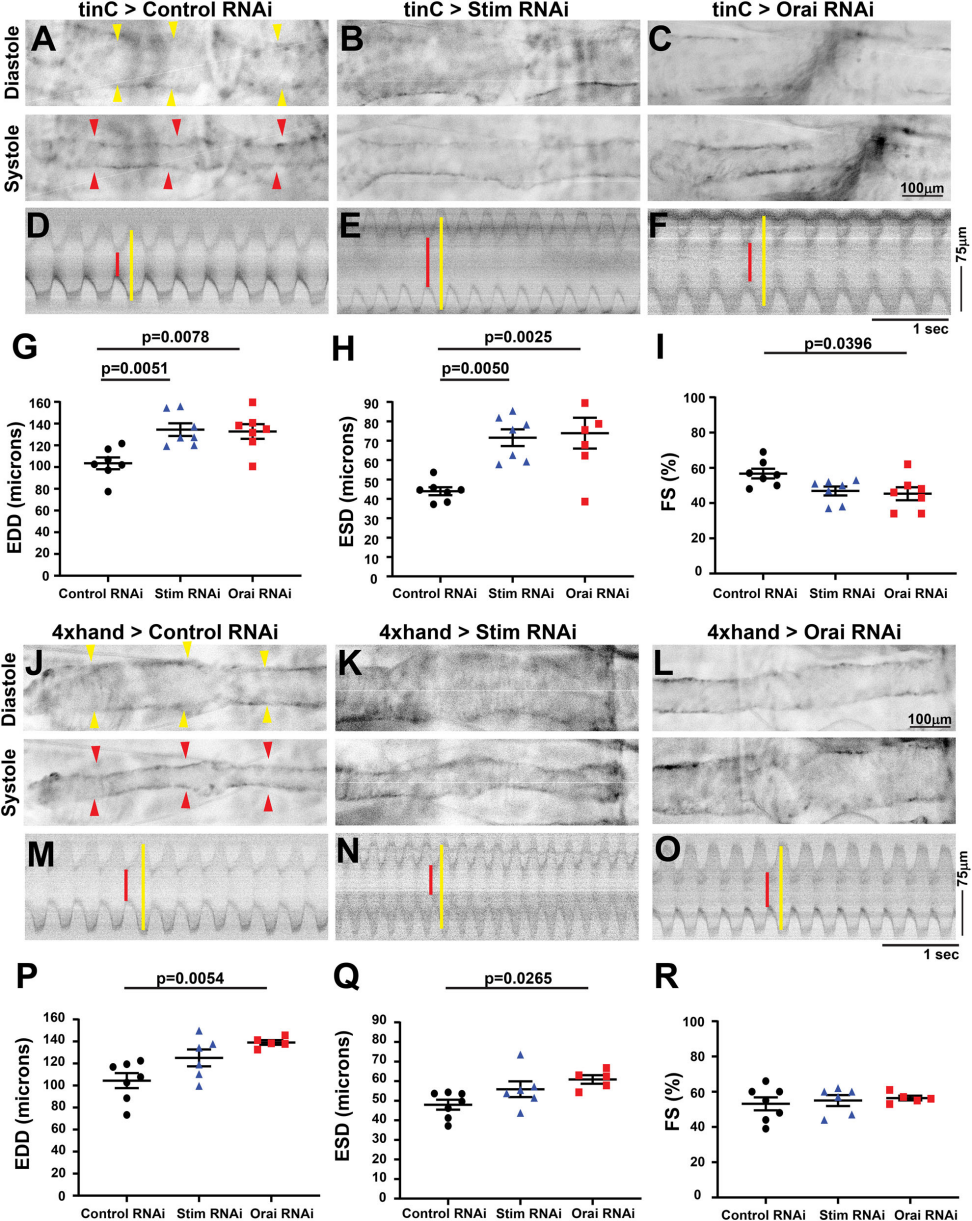
**Larval *Stim* and *Orai* suppressed hearts are significantly dilated.** (A–C) Representative longitudinal images from intravital fluorescence imaging of R94C02-tdTom-expressing third instar larval hearts with *tinC-GAL4*-driven non-targeting control, *Stim* and *Orai* RNAi during diastole (upper panels) and systole (lower panels). Images are presented in inverted grayscale for added clarity, due to low fluorescence intensity. Yellow arrowheads indicate heart in diastolic images and red arrowheads in systolic images. (D–F) Representative M-mode images from *tinC-GAL4*-driven non-targeting control, *Stim* and *Orai* RNAi hearts. Red lines depict systole and yellow lines diastole. EDD (G), ESD (H), FS (I) were calculated from M-mode recordings of third instar larva, and each symbol represents a measurement from a single animal. Bars indicate mean±s.e.m., and *P*-values were calculated from one-way ANOVA with Tukey's Multiple Comparison. (J–L) Representative longitudinal images from intravital fluorescence imaging of R94C02-tdTom expressing third instar larval hearts with *4xhand-GAL4* driven non-targeting control, *Stim* and *Orai* RNAi during diastole (upper panels) and systole (lower panels). Yellow arrowheads indicate heart in diastolic images and red arrowheads in systolic images. (M–O) Representative M-mode images from *4xhand-GAL4* driven non-targeting control, *Stim* and *Orai* RNAi hearts. Red lines depict systole and yellow lines diastole. EDD (P), ESD (Q), FS (R) were calculated from M-mode recordings of third instar larvae, and each symbol represents a measurement from a single animal. Bars indicate mean±s.e.m., and *P*-values were calculated from one-way ANOVA with Tukey's Multiple Comparisons Test.

Previous studies have demonstrated that cardiomyocyte-specific STIM1 suppression in mice results in age-dependent cardiomyopathy and heart failure in adults (Collins et al., 2014; Parks et al., 2016), whereas severe heart failure was evident during embryogenesis in zebrafish with Orai1 suppression (Volkers et al., 2012). Therefore, we determined whether cardiomyopathy caused by SOCE suppression in *Drosophila* is also age-dependent by analyzing contractility in larvae, prior to the adult life cycle stage. OCT has previously been used to analyze larval heart contractility (Bradu et al., 2009; Choma et al., 2011; Weismann et al., 2019); however, we have found on our system that light scattering caused by the high fat content of larvae significantly interferes with OCT analysis. We therefore analyzed larval contractility using intravital fluorescence imaging of animals with cardiomyocyte-specific tdTomato (CM-tdTom) expression (Klassen et al., 2017). Importantly, analysis of adult heart contractility by intravital CM-tdTom imaging yielded similar results to our OCT analysis, with significantly larger EDD and ESD and reduced FS in *tinC-GAL4* driven *Stim* RNAi hearts compared to controls (Fig. S3, Table S1, Movies 1 and 2). Similar to adults, larvae with *tinC-GAL4* driven *Stim* and *Orai* RNAi exhibited significantly increased EDD and ESD compared to non-targeting RNAi controls (Fig. 2A–H; Movies 3 and 4), and this resulted in significantly decreased FS for *Orai*, though not for *Stim* RNAi animals (Fig. 2I). A small though significant decrease in heart rate was also observed in *Orai* but not *Stim* RNAi larvae (Fig. S2C). These results suggest that reduced cardiac output due to SOCE suppression is already evident in larval stages of *Drosophila* development, and thus is not exclusively an age-dependent effect in the adult heart. Larval heart contractility was also evaluated with *4xhand-GAL4* driven *Stim* and *Orai* RNAi and although this resulted in modest increases in ESD and EDD, FS was unchanged compared to controls (Fig. 2J–R). It is possible that *4xhand-GAL4* does not drive RNAi expression as strongly in the larval heart as does *tinC-GAL4*, as has previously been noted (Lee et al., 2017), thus explaining the weaker effects of *4xhand-GAL4* driven *Stim* and *Orai* RNAi on larval heart contractility. Because of this, the *4xhand-GAL4* driver was not used for further larval heart analysis.

### *Stim* and *Orai* suppression disrupts myofibril organization

**Fig. 3.**
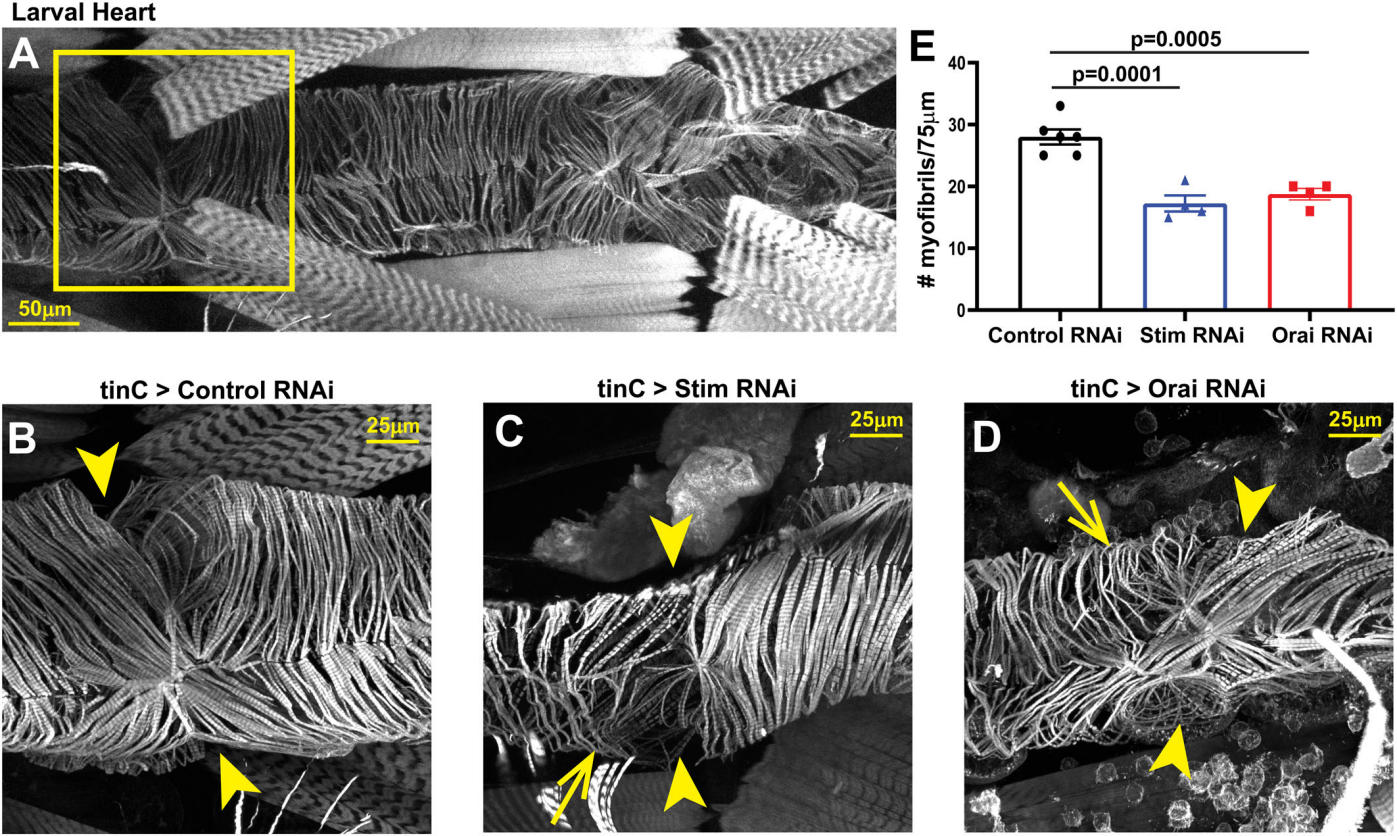
***Stim* and *Orai* suppression results in myofibril disorganization in larval hearts.** (A) Representative image of a control third instar larval heart stained with Phalloidin to visualize actin. The box denotes the region shown in high-resolution images. (B–D) Representative high-resolution images of the region around the second ostium of third instar larval hearts with *tinC-GAL4* driven non-targeting control, *Stim* and *Orai* RNAi. Arrowheads point to ostia, and arrows point to regions with disorganized myofibrils. (E) Plot of the total number of myofibrils per 75 µm in larval hearts with *tinC-GAL4* driven non-targeting control, *Stim* and *Orai* RNAi. Each symbol represents a measurement from a single heart, and data are mean±s.e.m. *P*-values were calculated from one-way ANOVA with Tukey's Multiple Comparisons Test.

**Fig. 4.**
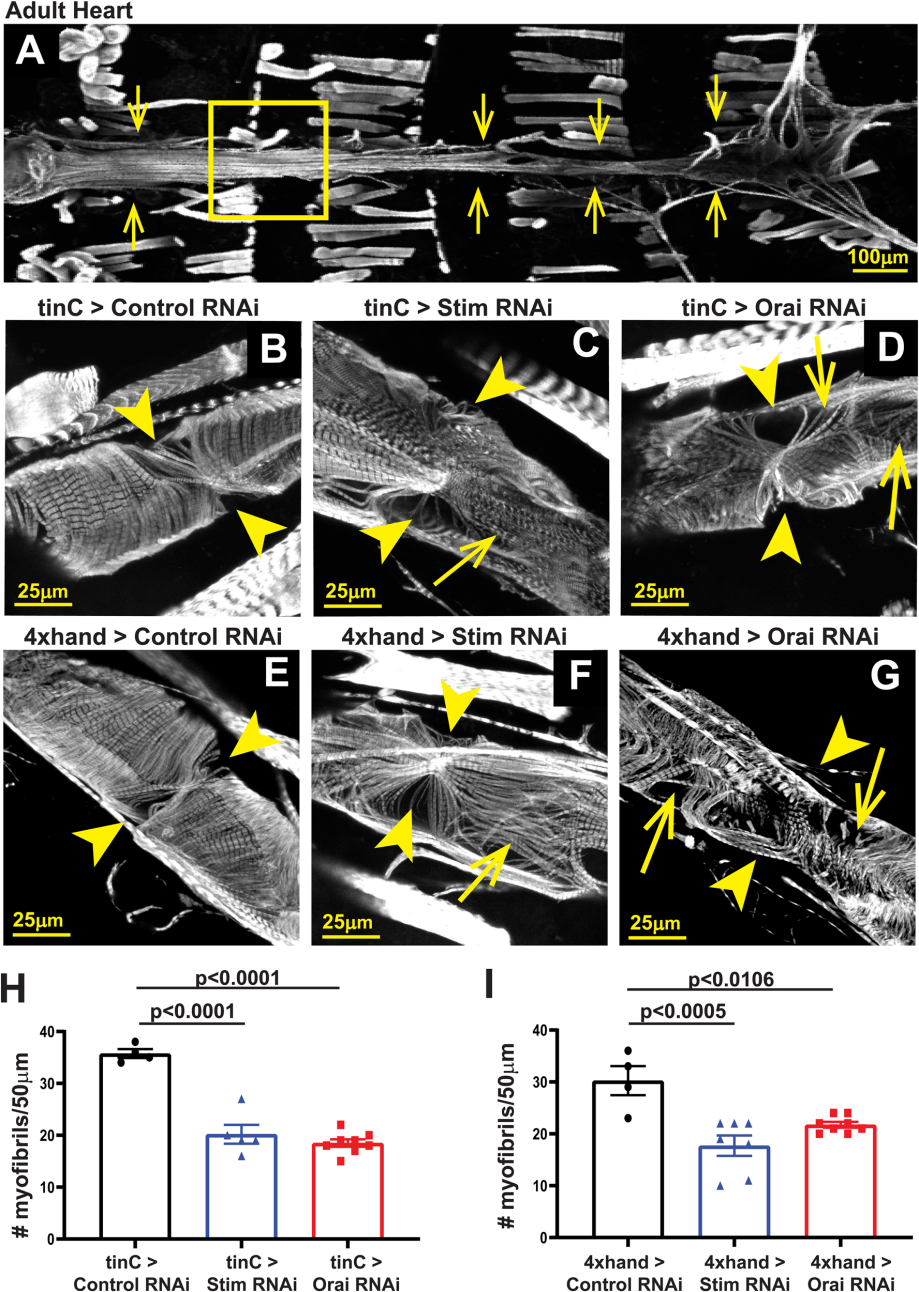
***Stim* and *Orai* suppression results in myofibril disorganization in adult hearts.** (A) Representative image of an adult control heart (indicated by arrows) stained with Phalloidin to visualize actin. The box denotes the region shown in the high-resolution images. (B–D) Representative high-resolution images of the region around the third ostium of 5-day-old adult male hearts with *tinC-GAL4* driven non-targeting control, *Stim* and *Orai* RNAi. Arrowheads point to ostia, and arrows point to regions with disorganized myofibrils. (E–G) Representative high-resolution images of the region around the third ostium of 5-day-old adult male hearts with *4xhand-GAL4* driven non-targeting control, *Stim* and *Orai* RNAi. Arrowheads point to ostia, and arrows point to regions with disorganized myofibrils. (H) Plot of the total number of myofibrils per 50 µm in adult hearts with *tinC-GAL4* driven non-targeting control, *Stim* and *Orai* RNAi. Each symbol represents a measurement from a single heart, and data are mean±s.e.m. *P*-values were calculated from one-way ANOVA with Tukey's Multiple Comparisons Test. (I) Plot of the total number of myofibrils per 50 µm in adult hearts with *4xhand-GAL4* driven non-targeting control, *Stim* and *Orai* RNAi. Each symbol represents a measurement from a single heart, and data are mean±s.e.m. *P*-values were calculated from one-way ANOVA with Tukey's Multiple Comparisons Test.

Disrupted myofibril organization is a common feature of dilated cardiomyopathy in mammalian (Garfinkel et al., 2018; Sussman et al., 1998) and *Drosophila* (Ocorr et al., 2007, 2014; Wolf et al., 2006) hearts. We therefore determined whether SOCE-suppressed hearts similarly exhibit disorganized myofibrils. As shown in Fig. 3A and B, hearts from *tinC-GAL4* driven control RNAi larvae exhibited evenly spaced myofibrils that were oriented circularly around the heart tube as revealed by actin staining and confocal microscopy. In contrast, myofibrils in *tinC-GAL4*-driven *Stim* and *Orai* RNAi hearts were disorganized and more widely spaced compared to controls, as indicated by a nearly 45% reduction in myofibril density in *Stim* and *Orai* RNAi versus control hearts (Fig. 3C–E). In adult control animals, myofibrils again were densely packed and uniformly oriented circularly around the heart tube (Fig. 4A,B,E). In marked contrast, however, myofibrils in adult *tinC-GAL4*-driven *Stim* and *Orai* RNAi hearts were highly disorganized, with many myofibrils oriented parallel to the long axis of the heart (Fig. 4C,D). Myofibril density was also significantly reduced in *Stim* and *Orai* RNAi compared to control adult hearts (Fig. 4H). Similar results were also seen in adult hearts with *4xhand-GAL4* driven *Stim* and *Orai* RNAi, including myofibril disorganization and a significant decrease in myofibril density (Fig. 4F,G,I). These results suggest that myofibril remodeling as a result of SOCE suppression may contribute to the aberrant contractility underlying dilated cardiomyopathy in these animals.

### Heart-specific *Stim* and *Orai* suppression reduces animal viability

**Fig. 5.**
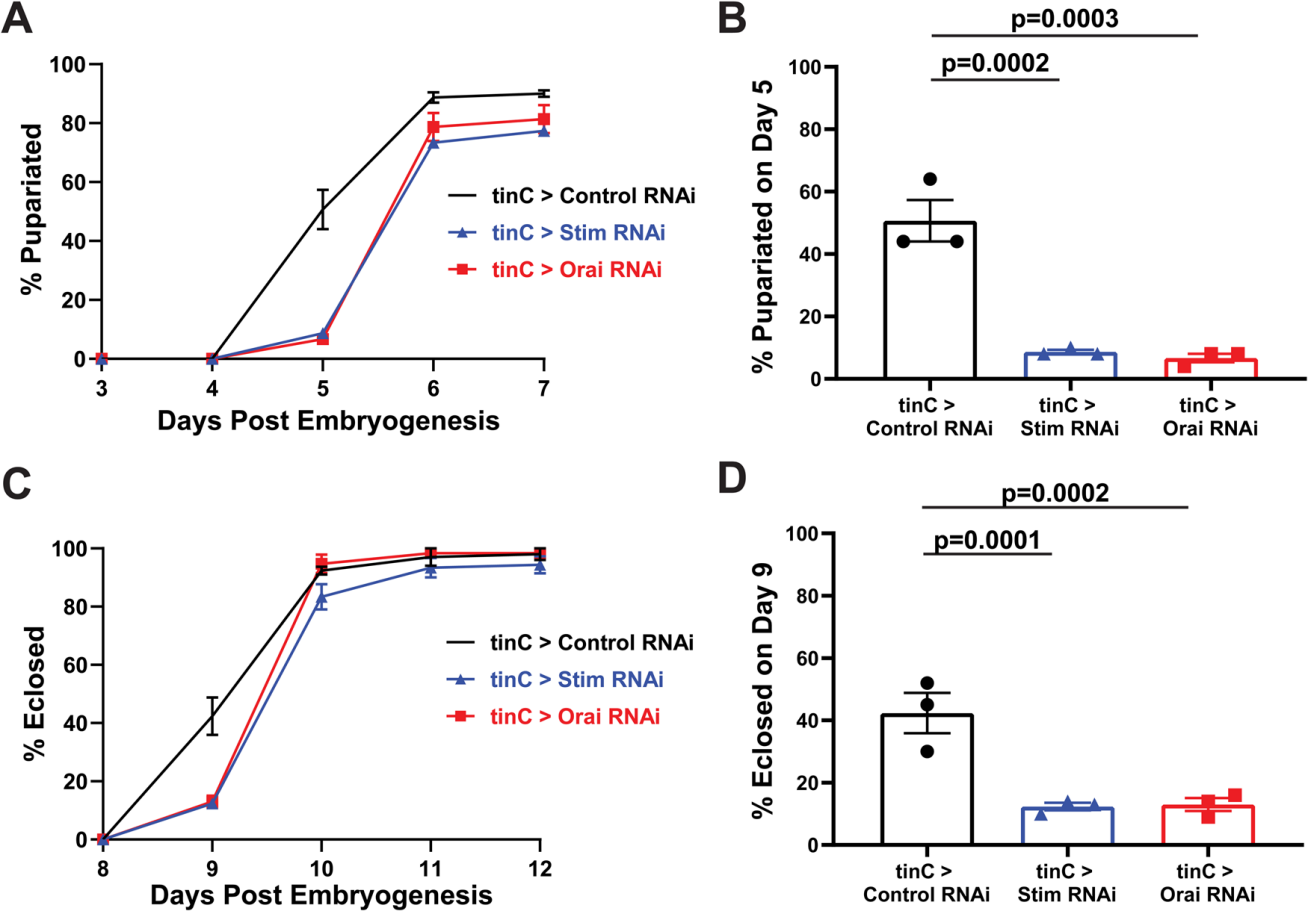
**Heart specific *Stim* and *Orai* suppression delays post-embryonic animal development.** (A) Plot of the percent of larvae that pupariated on each of the indicated days post-embryogenesis for *tinC-GAL4* driven *Stim*, *Orai* and non-targeting control RNAi. Data are mean±s.e.m. from three independent experiments with 25–50 animals per experimental group. (B) Comparison of percent pupariated on day 5 post-embryogenesis for *tinC-GAL4* driven *Stim*, *Orai* and non-targeting control RNAi from three independent replicates. *P*-values calculated from one-way ANOVA with Tukey's Multiple Comparisons Test. (C) Plot of the percent of pupae that eclosed on each of the indicated days post-embryogenesis for *tinC-GAL4* driven *Stim*, *Orai* and non-targeting control RNAi. Data are mean±s.e.m. from three independent experiments with 25–50 animals per experimental group. (D) Comparison of percent eclosed on day 9 post-embryogenesis for *tinC-GAL4* driven *Stim*, *Orai* and non-targeting control RNAi from three independent replicates. *P*-values calculated from one-way ANOVA with Tukey's Multiple Comparisons Test.

We next determined whether the functional defects in *Stim* and *Orai* suppressed hearts affect animal health and viability. In support of this, larval development was significantly prolonged in animals with *tinC-GAL4*-driven *Stim* and *Orai* RNAi, with nearly 86% fewer *Stim* and *Orai* RNAi animals having undergone pupariation by day 5 post-embryogenesis compared to controls (Fig. 5A,B). Numbers of pupariated *Stim* and *Orai* RNAi animals were indistinguishable from controls by day 6 post-embryogenesis, indicating that the overall delay in pupariation was approximately 24 h and that significant larval lethality was not observed. Eclosion, when adult animals emerge from their pupal cases, was also delayed by about 24 h in *tinC-GAL4*-driven *Stim* and *Orai* animals compared to controls, showing that development was not further prolonged during pupal metamorphosis (Fig. 5C–D). These results suggest that the contractile deficits caused by *Stim* and *Orai* suppression in the larval heart alter developmental physiology of the animal. Speculatively, reduced larval heart contractility may limit nutrient availability to developing tissues or reduce circulation of the hormone ecdysone that regulates larval developmental timing.

**Fig. 6.**
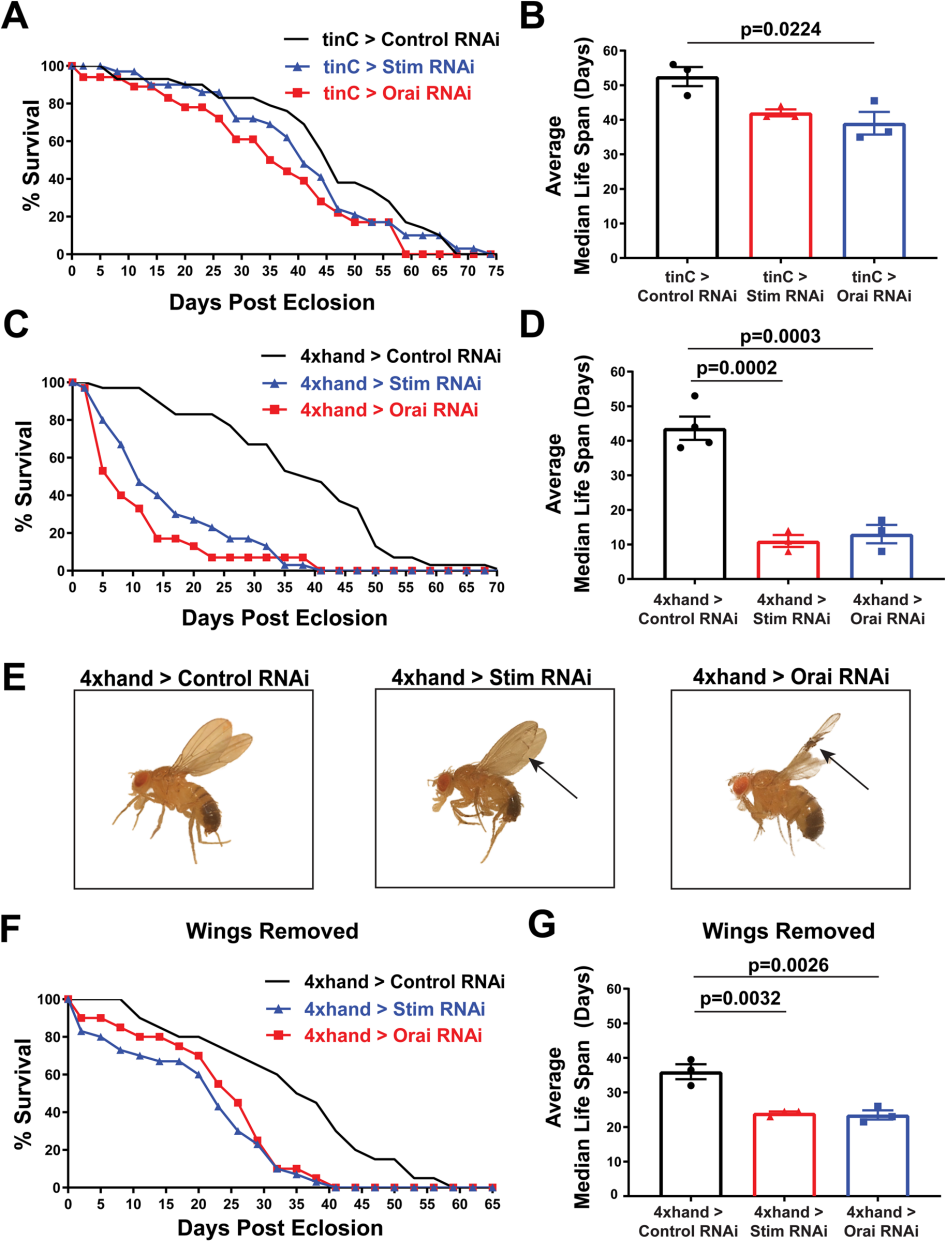
**Heart specific *Stim* and *Orai* suppression results in early adult lethality.** (A) Representative survival curves in days post-eclosion for adult males with *tinC-GAL4* driven *Stim*, *Orai* and non-targeting control RNAi (*n*=30 animals per group). (B) Plot of the average adult male median lifespan (MLS), calculated from three independent survival curves, for *tinC-GAL4* driven *Stim*, *Orai* and non-targeting control RNAi. Error bars represent s.e.m., and *P*-values were calculated from one-way ANOVA with Tukey's Multiple Comparisons Test. (C) Representative survival curves in days post-eclosion for adult males with *4xhand-GAL4* driven *Stim*, *Orai* and non-targeting control RNAi (*n*=30 animals per group). (D) Plot of the average adult MLS, calculated from three independent survival curves, for *4xhand-GAL4* driven *Stim, Orai* and non-targeting control RNAi. Error bars represent s.e.m., and *P*-values were calculated from one-way ANOVA with Tukey's Multiple Comparisons Test. (E) Representative images of adult males with *4xhand-GAL4* driven *Stim*, *Orai* and non-targeting control RNAi on the day of eclosion. Note the blistered wing on the *Stim* RNAi animal and the severely damaged and frayed wings on the *Orai* RNAi animal (indicated by arrows). (F) Representative survival curves in days post-eclosion for adult males with *4xhand-GAL4* driven *Stim*, *Orai* and non-targeting control RNAi, with their wings removed on day of eclosion (*n*=30 animals per group). (G) Plot of the average adult MLS, calculated from three independent survival curves, for *4xhand-GAL4* driven *Stim*, *Orai* and non-targeting control RNAi animals with their wings removed on the day of eclosion. Error bars represent s.e.m., and *P*-values were calculated from one-way ANOVA with Tukey's Multiple Comparisons Test.

We then analyzed adult lifespan to determine whether heart dysfunction resulting from *Stim* and *Orai* suppression affects adult viability. Males with *tinC-GAL4*-driven *Orai* RNAi died significantly earlier than those with non-targeting control RNAi, as indicated by a significant reduction of approximately 13 days in average median lifespan for *Orai* RNAi versus control animals (Fig. 6A,B). Animals with *tinC-GAL4*-driven *Stim* RNAi also exhibited an approximately 11 day reduction in average median lifespan compared to controls, though this difference was not statistically significant (Fig. 6A,B). More profound effects on adult lifespan were observed with *4xhand-GAL4*-driven *Stim* and *Orai* RNAi, as the average median lifespan of adult males was 11±1.73 and 13±2.65 days for *Stim* and *Orai* RNAi, respectively, compared to 43.64±3.38 days for controls (Fig. 6C–D; mean±s.e.m.). Thus, whereas *Stim* and *Orai* suppression driven by *tinC-GAL4* resulted in greater deficiencies in heart contractility, suppression driven by *4xhand-GAL4* resulted in greater reductions in adult lifespan. This discrepancy may result from broader tissue distribution of *4xhand-GAL4* expression in adults compared to *tinC-GAL4*, and thus *4xhand-GAL4*-driven RNAi may have effects on animal survival independent of heart function. Consistent with this, many *4xhand-GAL4*-driven *Stim* and *Orai* RNAi adults exhibited severe wing damage, including blistering and tearing of large sections of the wings (Fig. 6E). This is likely due to *4xhand-GAL4*-driven RNAi expression in wing hearts, which are muscles of cardiac origin that circulate hemolymph throughout the wings (Tögel et al., 2013, 2008). Interestingly, this suggests that SOCE is also required for proper wing heart function, though we did not investigate this further. However, we did consider the possibility that wing damage may have contributed to the early lethality of the *4xhand-GAL4*-driven RNAi animals by causing them to become stuck in the food or on the vials. To test this and more clearly determine whether lethality is attributable to dysfunction of the primary heart, we repeated adult survival experiments with *4xhand-GAL4*-driven RNAi animals whose wings were removed at the time of eclosion. As shown in Fig. 6F and G, wingless *4xhand-GAL4*-driven *Stim* and *Orai* RNAi animals indeed survived longer than those with wings (compare to Fig. 6C,D), but these animals still died significantly earlier than corresponding wingless controls. Importantly, the approximately 12-day reduction in average median lifespan of wingless *4xhand-GAL4*-driven *Stim* and *Orai* RNAi animals was similar to that of *tinC-GAL4*-driven RNAi animals, demonstrating consistent early lethality due to abnormal heart function. Collectively, these results suggest that heart dysfunction caused by SOCE suppression pathologically impairs developmental physiology and longevity in *Drosophila*.

## DISCUSSION

The critical importance of proper Ca^2+^ homeostasis and transport in cardiomyocytes is accentuated by the fact that nearly all cardiomyopathies involve Ca^2+^ dysregulation. Clear understanding of the cellular and molecular mechanisms that regulate cardiomyocyte Ca^2+^ handling is therefore fundamental to unraveling the complexities of cardiac pathophysiology. Our results demonstrate that SOCE is an essential component of cardiomyocyte Ca^2+^ physiology in *Drosophila*, because suppression of the key molecular components of the SOCE pathway, *Stim* and *Orai*, resulted in contractile deficiencies that are consistent with dilated cardiomyopathy. Concomitant with contractility defects, SOCE-suppressed hearts also exhibited disrupted myofibril organization and density, a common feature of genetic cardiomyopathy models. Moreover, our results show that reduced functional output of *Stim*- and *Orai*-suppressed hearts significantly impaired overall animal physiology and health, as these animals developed significantly more slowly and died earlier than controls. Importantly, our findings are largely consistent with those from mice and zebrafish in which STIM1 or Orai1, respectively, were suppressed (Collins et al., 2014; Volkers et al., 2012), strongly suggesting that SOCE has essential, cardiomyocyte-specific functions that are highly conserved across species.

Our current study augments previous findings showing cardiomyocyte-specific functions of STIM and Orai proteins in new and important ways. Most notably, our results are the first to demonstrate that suppression of both *Stim* and *Orai* in the same animal model results in nearly identical cardiac phenotypes suggesting that these proteins are both required and function together to regulate SOCE in cardiomyocytes. This is significant because previous studies in mammalian cardiomyocytes have implicated Orai1-independent targets of STIM1 including canonical transient receptor potential (TrpC) channels (Ohba et al., 2009) and L-type Ca^2+^ channels (Correll et al., 2015). While our results do not rule out the possibility of additional functional partners of STIM and Orai, particularly in vertebrate species, they do strongly suggest that Ca^2+^ influx mediated specifically by STIM-operated Orai channels is essential for proper cardiomyocyte physiology. This conclusion is further substantiated by the fact that *Drosophila* express single *Stim* and *Orai* isoforms. Thus, we can effectively target the SOCE pathway by suppressing only *Stim* or *Orai*, without possible compensation by other isoforms such as STIM2, Orai2, or Orai3. Moving forward, utilizing these advantages of the *Drosophila* model will allow us to investigate the functional roles of SOCE in cardiomyocytes using genetic tools that are largely unattainable with vertebrate models, including powerful genetic screens.

Despite several recent studies demonstrating that SOCE is required for normal, healthy heart function, the specific cellular processes regulated by SOCE in cardiomyocytes have not been clearly defined. Maintenance of SR Ca^2+^ stores is a seemingly likely function of SOCE that would broadly impact cardiomyocyte physiology. For example, decreased SR Ca^2+^ content in the absence of SOCE may reduce RyR-dependent Ca^2+^ release that drives contraction, and would be consistent with the severe systolic dysfunction that we and others have observed in STIM- and Orai-suppressed hearts. In this case, the myofibril disorganization observed in SOCE-suppressed hearts may be the result of decompensation in response to reduced contractile force generation (Houser and Margulies, 2003). However, direct analyses of SR Ca^2+^ store content in STIM- or Orai-depleted cardiomyocytes have yielded conflicting results. For example, siRNA knockdown of STIM1 in freshly isolated neonatal rat ventricular myocytes led to a significant reduction of the caffeine-releasable SR Ca^2+^ pools (Voelkers et al., 2010), whereas SR Ca^2+^ store content was unchanged in adult cardiomyocytes isolated from STIM1-deleted mouse hearts compared to controls despite clear indication of dilated cardiomyopathy (Parks et al., 2016). This suggests that SR Ca^2+^ store depletion may not be a direct cause of heart failure when SOCE is suppressed. However, an independent analysis of STIM1 deficiency-associated dilated cardiomyopathy in mice demonstrated significantly upregulated expression of ER stress response factors (Collins et al., 2014), possibly reflecting mild but chronic Ca^2+^ store depletion that may be difficult to measure in acutely isolated cells. Reorganization of cytoarchitecture is a common feature of the ER stress response mechanism, and therefore an important possibility in SOCE-suppressed cardiomyocytes is that ER stress leads to myofibril disorganization that contributes to impaired contractility and ultimately heart failure.

It is also possible that SOCE has specific signaling functions in cardiomyocytes that are required for heart development or tissue homeostasis. The best characterized signaling function of SOCE across cell and tissue types involves calcineurin-mediated dephosphorylation and activation of transcription factors such as NFAT (Lacruz and Feske, 2015). Overwhelming evidence has established that upregulated calcineurin-NFAT signaling is an essential mechanism of cardiac hypertrophy pathogenesis (Tham et al., 2015), and SOCE upregulation also results in cardiac hypertrophy (Benard et al., 2016; Hulot et al., 2011; Parks et al., 2016) and enhanced calcineurin activity (Luo et al., 2012). Collectively, these findings suggest that calcineurin is a key cardiomyocyte target of SOCE in the pathological, upregulated state. However, whether calcineurin or other signaling factors are essential targets of SOCE in normal heart physiology has not been determined. Arguing against a signaling role for SOCE specifically during heart development, STIM1-knockout mouse hearts do not exhibit altered phenotypes at early postnatal stages (Collins et al., 2014), and we also did not observe effects on *Drosophila* embryogenesis with heart-specific *Stim* or *Orai* suppression (data not shown). Additionally, cardiomyopathy in STIM1-knockout mouse hearts was progressive and age-dependent (Collins et al., 2014), further suggesting that any signaling functions of SOCE are likely to be homeostatic rather than developmental. Our findings in *Drosophila* indicate that cardiomyopathy due to *Stim* and *Orai* suppression is already evident in larvae, early in the fly life cycle. This may also reflect post-developmental, homeostatic functions of SOCE that when suppressed, manifest earlier in flies due to their faster lifecycle compared to mice. Clearly, substantial work is still required to identify the specific functional roles of SOCE in physiological heart function, and genetic tools combined with *in vivo* analyses of heart physiology and Ca^2+^ dynamics in *Drosophila* will allow us to address this in novel and significant ways.

Our study is the first to demonstrate the use of intravital fluorescence imaging of heart contractility in *Drosophila* larvae, an important lifecycle stage that involves rapid animal growth and high nutritional demands. This is significant because larval heart contractility has been difficult to analyze using methods commonly employed for adults, such as OCT and direct imaging of dissected preparations. For example, the high fat content of larvae causes light scattering that can obscure OCT imaging, though some OCT analyses of larval heart function have been carried out successfully (Bradu et al., 2009; Choma et al., 2011; Weismann et al., 2019). In addition, partial dissections that faithfully preserve heart function can be challenging because the larval heart is extremely delicate and poorly attached to the body wall. Thus, intravital fluorescence imaging of larval hearts is a versatile and widely accessible method that is also amenable to longitudinal analysis. We validated our intravital imaging approach by confirming significantly increased ESD and EDD and decreased FS values in adult *Stim* RNAi versus control hearts, similar to our results from the more established OCT method. However, it was notable that even in controls, ESD and EDD values were substantially larger with intravital imaging compared to OCT measurements (Table S1). This may be due in part to better spatial resolution of the heart walls with intravital fluorescence imaging compared to OCT. Moreover, OCT measures the dorsal to ventral movement of the heart walls, whereas intravital fluorescence imaging measures lateral movement. This important distinction may also account for the differences we observed with the two methods. Importantly, our fluorescence imaging measurements of adult heart ESD, EDD and FS were very similar to those from an earlier study that first described the CM-tdTom model for intravital fluorescence imaging (Klassen et al., 2017), further validating the accuracy of our measurements.

Another notable finding of our study was that there were several key differences in the results of *Stim* and *Orai* RNAi driven with *tinC-GAL4* compared to *4xhand-GAL4*, both of which are commonly used as heart-specific *GAL4* drivers in *Drosophila* studies. Most strikingly, *4xhand-GAL4* but not *tinC-GAL4* driven RNAi resulted in significant effects on adult wing structure and integrity that were likely caused by defective wing heart function, given that *hand* but not *tinman* (the genes from which the respective *GAL4* drivers are derived) is expressed in wing heart myocytes (Tögel et al., 2013, 2008). Importantly, we found that the wing defects caused by *4xhand-GAL4* driven *Stim* and *Orai* RNAi significantly shortened animal lifespan, independent of effects on contractility of the primary adult heart. This illustrates that caution is warranted when attributing systemic phenotypes, such as premature lethality, to dysfunction of the primary *Drosophila* heart when using these *GAL4* drivers.

In conclusion, our results demonstrate that SOCE mediated by STIM and Orai is essential for proper function of the *Drosophila* heart, and add to a growing number of studies that collectively suggest that SOCE has highly conserved functional roles in cardiomyocytes across species. Paradoxically, it is still unknown whether loss-of-function mutations in *Stim1* or *Orai1* result in heart defects or cardiomyopathies in humans. This is likely due to the fact that individuals homozygous for these mutations die from immunodeficiency in infancy or early childhood, before adverse heart phenotypes may fully manifest (Rosenberg et al., 2019). Therefore, animal model studies, including powerful genetic screens and *in vivo* analyses in *Drosophila*, will continue to be vital to our understanding of how alterations to SOCE result in or contribute to devastating cardiomyopathies and heart failure.

## MATERIALS AND METHODS

### Fly stocks

The following *Drosophila* stocks were obtained from the Bloomington *Drosophila* Stock Center: mCherry RNAi (35785; non-targeting control), GAL4 RNAi (35783; non-targeting control), *Stim* RNAi (27263), *Orai* RNAi (53333), and *act-GAL4* (3954). *tinC-GAL4* was obtained from Dr Manfred Frausch (Friedrich Alexander University), and *4xhand-GAL4* was from Dr Zhe Han (George Washington University School of Medicine). CM-tdTom flies were obtained from Dr Rolf Bodmer (Sanford Burnham Prebys Institute). Flies were maintained on standard cornmeal agar food, and all crosses were carried out at 25°C.

### Adult survival

Virgin female *tinC-GAL4* or *4xhand-GAL4* flies were crossed with male RNAi animals, and progeny were raised to adulthood at 25°C. On the day of eclosion, adult progeny were collected and separated based on sex into vials containing up to ten flies per vial, with a total of 20–30 flies per group. Flies were maintained at 25°C throughout the course of the experiments. Every 3 days, vials were checked for dead animals and surviving flies were transferred into new vials with fresh food. For wingless experiments, wings were removed immediately following eclosion on the day of adult collection. Kaplan–Meier survival curves and median lifespan were generated using GraphPad Prism.

### Developmental timing

Approximately 30–40 virgin female *tinC-GAL4* or *4xhand-GAL4* animals were mated with RNAi males for 3 days, at which time animals were transferred into egg laying chambers that consisted of a 100 ml plastic beaker with holes for air exchange affixed over a petri dish containing grape juice agar (Genesee Scientific). A dollop of yeast paste (active dry yeast mixed with water) was placed in the center of each grape juice agar plate as food. Animals were acclimated in the chambers for 24 h, and then transferred to new plates for 4 h at 25°C for timed egg laying. After removing the adults, plates with eggs were incubated at 25°C for an additional 24 h. Hatched larvae were then transferred to vials with standard fly food, with up to 30 larvae per vial, and maintained at 25°C over the course of the experiment. Vials were checked each day, and the numbers of newly formed pupae and eclosed adults were recorded.

### RNA Isolation and RT-qPCR

Twenty to 30 first instar larvae were collected in 20 µl cold Trizol and manually crushed with a pestle. Additional Trizol was then added to a total volume of 500 µl, 100 µl chloroform was added, and the aqueous layer containing extracted RNA was isolated. Extracted RNA was further purified with the RNeasy kit (Qiagen) and converted to cDNA using an S1000 Thermo Cycler (Bio-Rad) with high capacity cDNA Reverse Transcription kit (Thermo Fisher Scientific). Real-time quantitative polymerase chain reaction (RT-qPCR) was performed on a StepOnePlus RT-qPCR machine (Applied Biosystems) with each reaction consisting of triplicate samples containing iTaq Universal Probes Supermix (Bio-Rad), pre-validated 6-carboxyfluorescein (FAM)-labeled TaqMan probes (Applied Biosystems) against *Stim*, *Orai* and *RPL32* (housekeeping gene), and template cDNA diluted per the manufacturer's instructions. For quantification, triplicate cycle threshold (Ct) values were averaged and normalized to the *RPL32* Ct value to calculate ΔCt. The Δ(ΔCt) was determined by subtracting the control RNAi ΔCt value from the experimental ΔCt value, and fold changes expressed as 2^−Δ(ΔCt)^. Fold changes are expressed as a percentage of expression compared to non-targeting RNAi control.

### Heart dissection, staining and confocal imaging

Hearts from third instar larvae or 5-day-old adults were dissected and fixed as previously described (Alayari et al., 2009). In brief, for adults the ventral abdomens and underlying tissues were removed to expose the contracting heart while bathed in oxygenated artificial *Drosophila* hemolymph [ADH; 108 mM NaCl, 5 mM KCl, 2 mM CaCl_2_, 8 mM MgCl_2_, 1 mM NaH_2_PO_4_, 4 mM NaHCO_3_, 10 mM sucrose, 5 mM trehalose, and 5 mM HEPES (pH 7.1)]. Hearts were then fully relaxed by exchange with fresh ADH containing 10 mM EGTA. Larvae were pinned to Sylgard coated dishes at their anterior and posterior, and a slit was cut along the ventral midline in the presence of oxygenated ADH. Lateral cuts were then made along the sides of the animals, and the resulting cuticle flaps were pinned to expose the internal organs. The gut was removed to expose the beating hearts, which were then relaxed with ADH containing 10 mM EGTA. Dissected adult and larval hearts were both fixed for 20 min at room temperature in PBS containing 4% paraformaldehyde. Following three 10 min washes in PBS containing 0.1% Triton X-100 (PBSTx) with gentle rotation, the samples were incubated in PBSTx containing 1.0 µM Alexa Fluor 488 Phalloidin (Thermo Fisher Scientific) for 1 h at room temperature with gentle shaking. Samples were again washed three times in PBSTx at room temperature, and mounted on glass slides and coverslips with Vectashield (Vectashield Laboratories) as described previously (Alayari et al., 2009). Samples were imaged with a Nikon A1R confocal microscope using 10X, 0.45 N.A. and 40X, 1.3 NA objectives. Phalloidin was excited with a 488 nm laser. Z-stacks at 1 µm intervals were collected and images are presented as maximum intensity projections encompassing the whole heart for larvae or the dorsal half of the heart for adults to avoid the ventral layer of skeletal muscle. Myofibril density measurements on adults and third instar larval hearts were taken from the A2 segment of the heart. The number of myofibrils per 75 µm in larvae and 50 µm in adults was calculated manually.

### Optical coherence tomography (OCT)

Adult heart contractility was analyzed using a custom-built OCT apparatus as previously described (Wolf et al., 2006). In brief, 5-day-old males were briefly anesthetized with CO_2_, embedded in a soft gel support, and allowed to fully awaken based on body movement. Animals were first imaged in B-mode in the longitudinal orientation to identify the A1 segment of the heart chamber. They were then imaged transversely in M-mode for 3 s, and multiple M-modes were recorded for each fly. Animals were then re-imaged in B-mode to ensure proper orientation of the heart chamber. M-modes were processed in ImageJ by referencing to a 150 µm standard. EDD, ESD and heart rate were calculated directly from the processed M-mode traces. FS was calculated as [(EDD-ESD) / EDD]×100.

### Intravital fluorescence microscopy

Intravital fluorescence imaging of adult and third instar larval hearts was carried out using animals that express tdTomato under control of the cardiomyocyte-specific R94C02 enhancer element (Klassen et al., 2017). 5-day-old adult males were briefly anesthetized with CO_2_ and were then adhered dorsal side down to a glass coverslip with Norland Optical Adhesive that was then cured with a 48-watt UV LED light source (LKE) for 60 s. Animals were allowed to recover for 10 min prior to imaging. Third instar larvae were anesthetized by 1-min cold exposure at 4°C and were adhered ventral side down to a glass slide with superglue. Larvae were flanked on the glass slide by double-sided tape that was then used to adhere a glass coverslip. The heart of larval and adult animals was imaged through the dorsal cuticle at a rate of 200 frames per second (fps) for 10 s using an ORCA-Flash4.0 V3 sCMOS camera (Hamamatsu) on a Nikon Ti2 inverted microscope controlled with Nikon Elements software. Excitation light at 550 nm was provided by a Spectra-X illuminator (Lumencor) and emission was collected through a 555-635 band-pass filter. To generate M-modes, a 1-pixel wide line was drawn through the heart chamber in the A1 segment in adults and the A2 segment in larvae, and the fluorescence intensity along this line for the full time-course was plotted using ImageJ. EDD and ESD were calculated directly from the processed M-mode traces by manually measuring the distance between the heart walls at full relaxation and full constriction, respectively. An average of five measurements of EDD and ESD was calculated from each trace. Heart rate was calculated by manually counting the number of systoles over 10 s. FS was calculated as [(EDD-ESD)/EDD]×100.

### Statistical analyses

All statistical analyses were carried out using GraphPad Prism software. Contractility parameters and myofibril density measurements were analyzed by one-way ANOVA followed by Tukey's Multiple Comparisons Test. Differences were considered statistically significant at *P*<0.05.

## Supplementary Material

Supplementary information
