# Supplementary material for: Suppression of store-operated calcium entry causes dilated cardiomyopathy of the Drosophila heart
Source: Biol Open. 2020 Mar 11;9(3):bio049999. doi: 10.1242/bio.049999 (PMC7075072; doi:10.1242/bio.049999)
Supplement: Supplementary information [file biolopen-9-049999-s1.pdf]

Figure S1

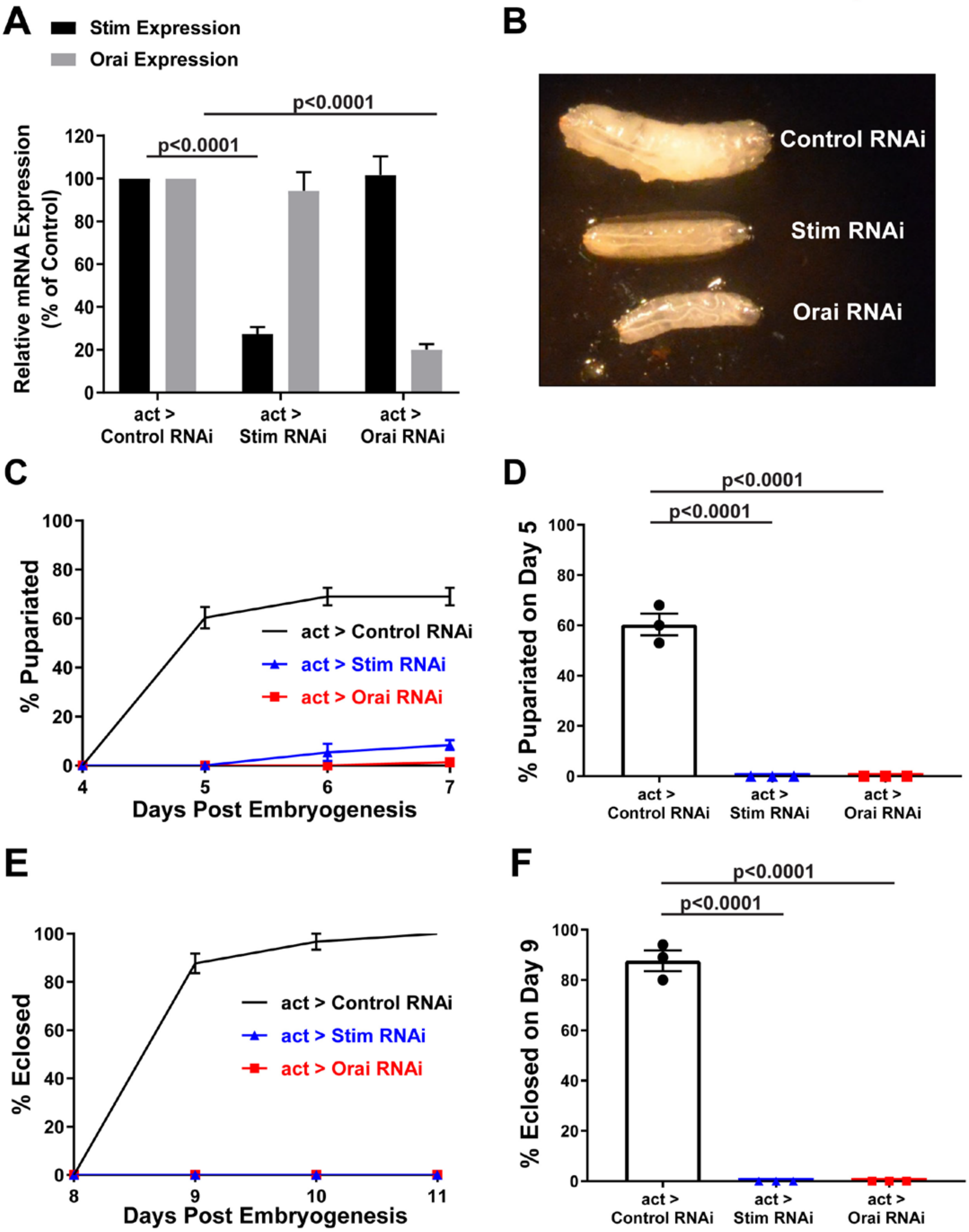

Biology Open • Supplementary information

Figure S1. ***Stim* and *Orai* RNAi are efficient and specific**

A. RT-qPCR analysis of relative *Stim* and *Orai* mRNA expression levels from first instar larvae with *act-GAL4* driven non-targeting control, *Stim*, and *Orai* RNAi. Data are represented as percent of non-targeting control RNAi (mean ± s.e.m. from three independent replicates; p-values were calculated from Two-way ANOVA with Tukey's Multiple Comparison). **B.** Representative images of third instar larvae with *act-GAL4* driven non-targeting control, *Stim*, and *Orai* RNAi. Note the significantly reduced size of *Stim* and *Orai* RNAi animals compared to controls. **C.** Plot of the percent of larvae that pupariated on each of the indicated days post-embryogenesis for *act-GAL4* driven *Stim*, *Orai*, and non-targeting control RNAi. Data are mean ± SEM from three independent experiments, with 25-50 animals per experimental group. **D.** Comparison of percent pupariated on day 5 post-embryogenesis for *act-GAL4* driven *Stim*, *Orai,* and non-targeting control RNAi from three independent experiments (p-values calculated from One-way ANOVA with Tukey's Multiple Comparisons Test). **E.** Plot of the percent of pupae that eclosed on each of the indicated days post-embryogenesis for *act-GAL4* driven *Stim*, *Orai,* and non-targeting control RNAi. Data are mean ± SEM from three independent experiments, with 25-50 animals per experimental group. **F.** Comparison of percent eclosed on day 9 post-embryogenesis for *act-GAL4* driven *Stim*, *Orai* and non-targeting control RNAi from three independent experiments (p-values calculated from One-way ANOVA with Tukey's Multiple Comparisons Test).

Biology Open • Supplementary information

Figure S2

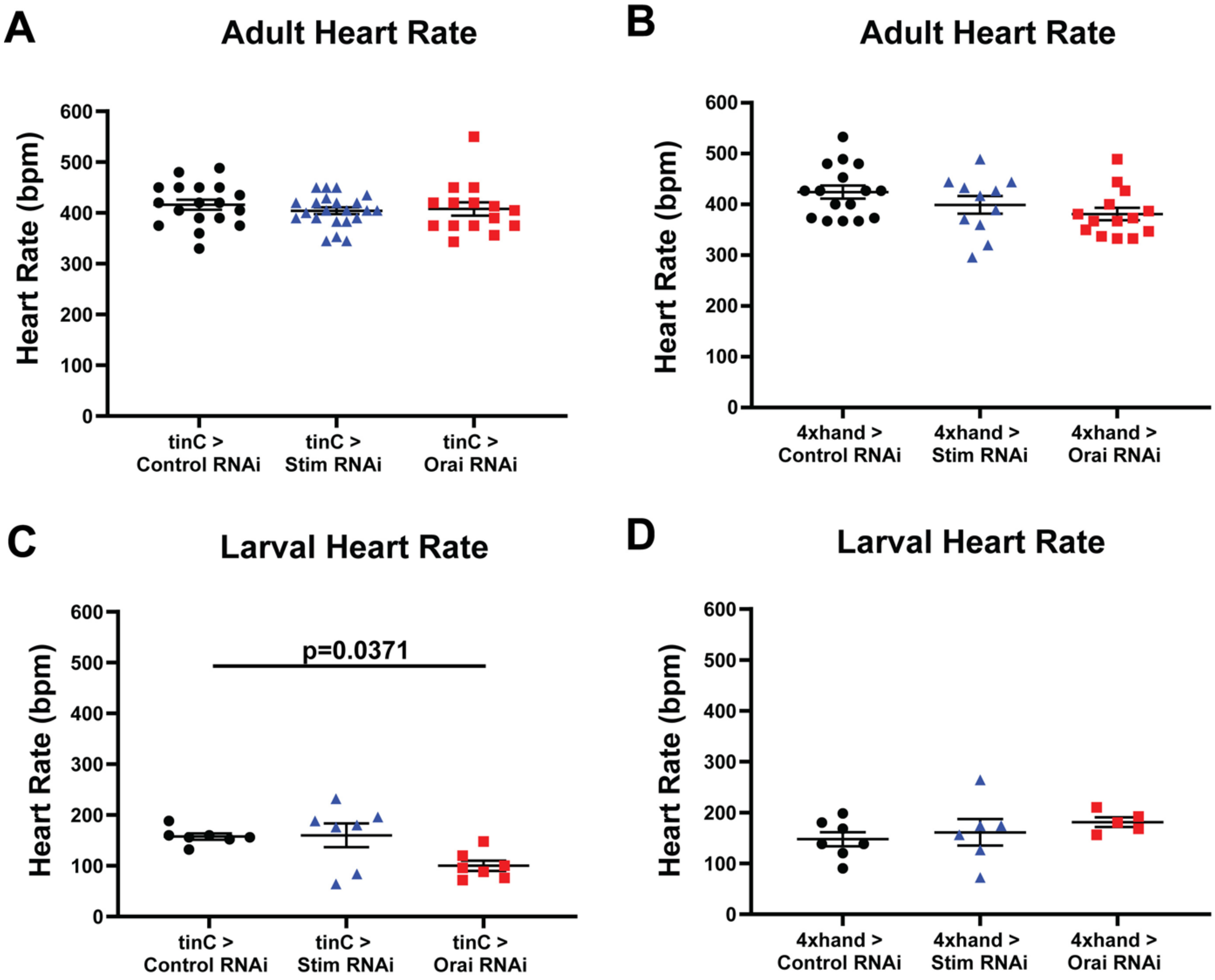

Biology Open • Supplementary information

Figure S2: Heart specific suppression of *Stim* and *Orai* does not affect adult or larval heart rate

Adult heart rate calculated as beats per min (bpm) from OCT imaging for *tinC-GAL4* (**A**) and *4xhand-GAL4* (**B**) driven non-targeting control, *Stim*, and *Orai* RNAi animals. Each symbol represents a single animal measurement; results were not significantly different (One-way ANOVA with Tukey's Multiple Comparisons Test). Third instar larval heart rate calculated as beats per min (bpm) from intravital fluorescence imaging for *tinC-GAL4* (**C**) and *4xhand-GAL4* (**D**) driven non-targeting control, *Stim* and *Orai* RNAi. Each symbol represents a single animal measurement; results were not significantly different except where indicated (p-value calculated from One-way ANOVA with Tukey's Multiple Comparison).

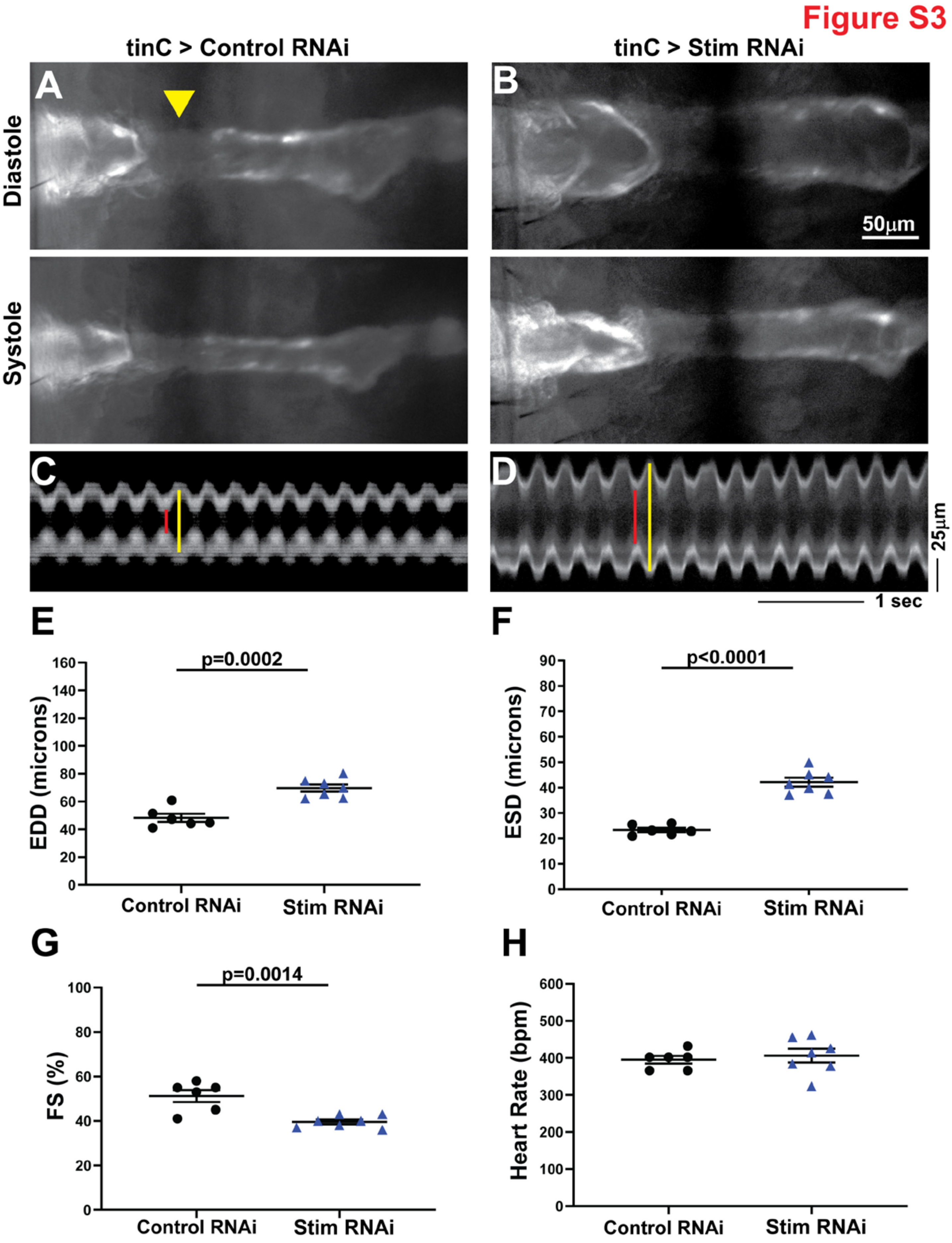

Biology Open • Supplementary information

Figure S3: Intravital fluorescence imaging confirms dilated cardiomyopathy in adult *Stim* and *Orai* suppressed hearts

A-B. Representative longitudinal B-mode images of intravital fluorescence imaging of R94C02-tdTom expressing five-day old adult male hearts with *tinC-GAL4* driven non-targeting control and *Stim* RNAi during diastole (upper panels) and systole (lower panels). Arrowhead points to region that is obscured by a dark abdominal stripe on the cuticle of the animal. **C-D.** Representative M-mode images from *tinC-GAL4* driven non-targeting control and *Stim* RNAi hearts, with red lines depicting systole and yellow lines diastole. EDD (**E**), ESD (**F**), FS (**G**), and heart rate (**H**) were calculated from M-mode recordings of five-day old males, and each symbol represents a measurement from a single animal. Bars indicate mean ± s.e.m., and p-values were calculated from unpaired t-tests.

Biology Open • Supplementary information

	**Adult OCT**	**Adult CM-tdTom**	**Larval CM-tdTom**
**End Diastolic Dimensions**	73.61 ± 2.54 µm	48.31 ± 2.89 µm	103 ± 5.41 µm
**End Systolic Dimensions**	8.89 ± 1.63 µm	23.32 ± 0.83 µm	43.92 ± 2.05 µm
**Fractional Shortening**	88.63 ± 2.04 %	51.17 ± 2.71 %	56.71 ± 2.76 %
**Heart Rate**	416.3 ± 9.87 bpm	395 ± 10.32 bpm	157.7 ± 6.23 bpm

Table S1. Comparison of contractility parameters measured from OCT and CM-tdTom intravital imaging analyses for control animals.

Table indicates mean ± s.e.m. from OCT (n = 18) and CM-tdTom intravital imaging (n = 6) of *tinC-GAL4* driven nontargeting control RNAi adult hearts, as well as *tinC-GAL4* driven nontargeting control RNAi larval hearts measured by CM-tdTom intravital imaging (n = 7).

Biology Open • Supplementary information

SUPPLEMENTARY MOVIES

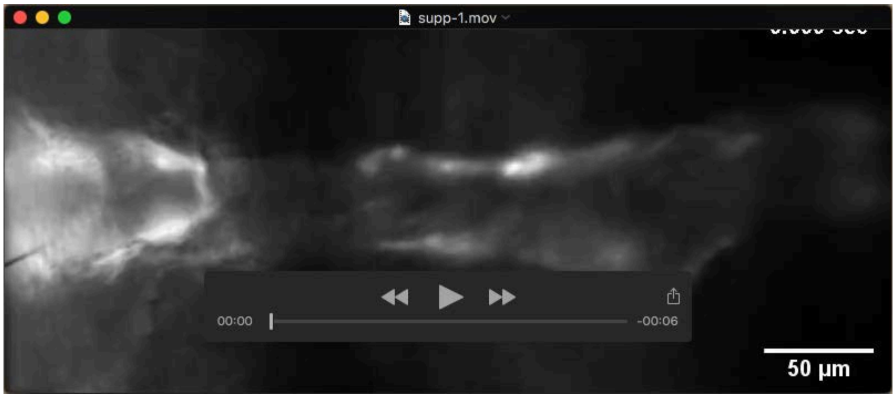

<u>Movie 1.</u> Full intravital imaging timelapse of the adult control heart shown in Figure S3. Images were acquired at a rate of 200 frames per second, and video playback is slowed to 100 frames per second to allow better visualization of contractions.

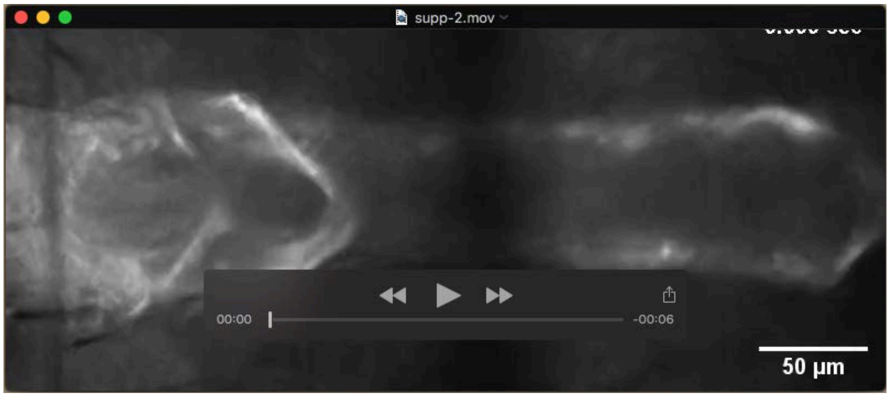

<u>Movie 2.</u> Full intravital imaging timelapse of the adult *Stim* RNAi heart shown in Figure S3. Images were acquired at a rate of 200 frames per second, and video playback is slowed to 100 frames per second to allow better visualization of contractions.

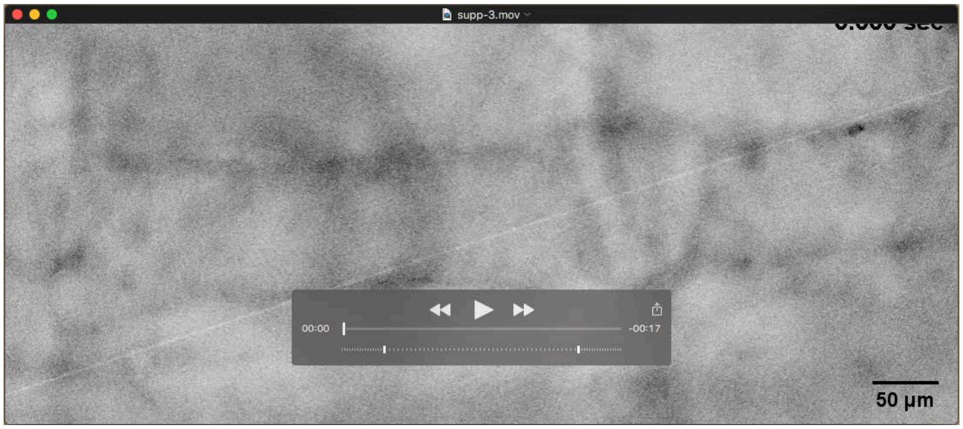

<u>Movie 3.</u> Full intravital imaging timelapse of the larval control heart shown in Figure 2A.

Images were acquired at a rate of 200 frames per second, and video playback is at full frame-rate.

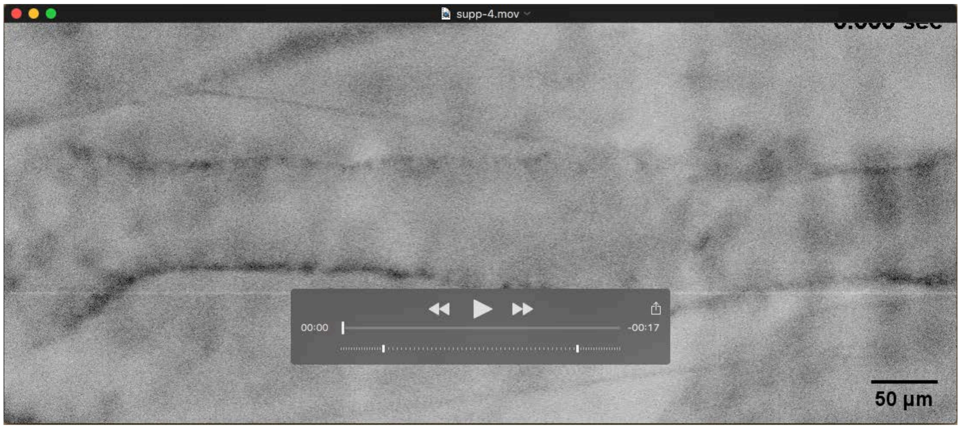

<u>Movie 4.</u> Full intravital imaging timelapse of the larval *Stim* RNAi heart shown in Figure 2B.

Images were acquired at a rate of 200 frames per second, and video playback is at full frame-rate.

Biology Open • Supplementary information
